# Cost-utility analysis of biologic disease-modifying antirheumatic drugs (bDMARDs), targeted synthetic DMARDs (tsDMARDs) and biosimilar DMARDs (bsDMARDs) combined with methotrexate for Thai rheumatoid arthritis patients with high disease activity

**DOI:** 10.1186/s12913-023-09595-1

**Published:** 2023-05-31

**Authors:** Juthamas Prawjaeng, Pattara Leelahavarong, Nuttakarn Budtarad, Songyot Pilasant, Chonticha Chanjam, Wanruchada Katchamart, Pongthorn Narongroeknawin, Tasanee Kitumnuaypong

**Affiliations:** 1 Siriraj Health Policy Unit, Faculty of Medicine Siriraj Hospital, Mahidol University, Bangkok, Thailand; 2 Health Intervention and Technology Assessment Program (HITAP), Ministry of Public Health, Nonthaburi, Thailand; 3 Siriraj Hospital, Faculty of Medicine Siriraj Hospital, Mahidol University, Bangkok, Thailand; 4 United States Agency for International Development (USAID) Regional Development Mission for Asia, Bangkok, Thailand; 5 Puey Ungphakorn School of Development Studies, Thammasat University, 99 Moo 18 Paholyothin Road, Klong Nueng, Klong Luang, Pathumthani, Thailand; 6 Division of Rheumatology, Department of Medicine, Faculty of Medicine Siriraj Hospital, Mahidol University, Bangkok, Thailand; 7 Division of Rheumatology, Department of Internal Medicine, Phramongkutklao Hospital and College of Medicine, Bangkok, Thailand; 8 Rheumatology Unit, Department of Medicine, Rajavithi Hospital, Bangkok, Thailand

**Keywords:** Economic evaluation, Cost-utility analysis, Disease-modifying antirheumatic drugs, Biologic DMARDs, Targeted synthetic DMARDs, Biosimilar DMARDs, Rheumatoid arthritis

## Abstract

**Background:**

New biologic disease-modifying antirheumatic drugs (bDMARDs), targeted synthetic DMARDs (tsDMARDs) and biosimilar DMARDs (bsDMARDs) all showed greater clinical benefits in the treatment of patients with rheumatoid arthritis (RA) with high disease activity, but imposed higher costs than standard treatment. This study evaluated the cost-effectiveness of 11 alternative treatment strategies for RA patients with high disease activity whose treatment with three conventional synthetic DMARDs (csDMARDs) failed.

**Methods:**

A Markov model was constructed using a societal perspective to estimate relevant costs and health outcomes in terms of quality-adjusted life years (QALYs) for a lifetime horizon (100 years), given a 3% annual discount. Alternative treatment strategies including five bDMARDs, two tsDMARDs, and four bsDMARDs in combination with methotrexate (MTX) were compared with the standard of care (SoC), i.e., cyclosporine and azathioprine. Direct and non-medical care costs were estimated by identifying the resources used, then multiplied by the standard costing menu in the year 2022. Utility and transitional probabilities were collected in three advanced tertiary hospitals. A network meta-analysis was used to estimate the efficacy of each treatment. Lifetime cost, QALYs and an incremental cost-effectiveness ratio were calculated and compared to the cost-effectiveness threshold of 160,000 THB per QALY gained (US $4,634, where 1 USD = 34.53 THB in 2022). Probabilistic and one-way sensitivity analyses were performed to estimate parameter uncertainties.

**Results:**

The bDMARDs, tsDMARDs or bsDMARDs combined with MTX provided 0.09 to 0.33 QALYs gained with additional costs of 550,986 to 2,096,744 THB (US $15,957 to $60,722) compared to the SoC. The ICER ranged from 2.3 to 8.1 million THB per QALY (US $65,935 to $234,996) compared to the SoC. None of these combinations was cost-effective in the Thai context. The results were sensitive to the mortality hazard ratio of patients with high disease activity.

**Conclusions:**

Combinations of MTX with either bDMARDs, tsDMARDs or bsDMARDs were not economically attractive compared to the standard practice. However, they reduced disease activity and improved patient quality of life. The price negotiation process for these treatments must be conducted to ensure their financial value and affordability before they are included in the pharmaceutical reimbursement list.

**Supplementary Information:**

The online version contains supplementary material available at 10.1186/s12913-023-09595-1.

## Introduction

Rheumatoid arthritis (RA) is a chronic progressive inflammatory disorder of unknown etiology characterized by symmetric polyarticular joint involvement that can cause joint destruction. The goals of treatment are to induce complete remission or decrease disease activity. Additional goals include controlling joint pain, delaying joint damage that leads to disability, and maintaining or improving both the ability to function in daily activities and the patient’s quality of life [1]. In 2015, the incidence and prevalence of RA were estimated to be 0.15% and 1.6% of the total Thai population, respectively [2]. Ineffective RA treatment can cause high disease activity, leading to a 2.43-fold increase in mortality rate compared to RA patients with low disease activity (hazard ratio 2.43; 95% CI 1.64 to 3.61) [3]. It can also lead to lifelong disability, which reduces quality of life and reduces productivity [4]. According to the Thai RA clinical practice guidelines, conventional synthetic disease-modifying antirheumatic drugs (csDMARDs), anti-inflammatory drugs, analgesics, and low-dose corticosteroids are recommended as first-line therapies. Treatments with csDMARDs such as methotrexate (MTX), sulfasalazine (SSZ), chloroquine (CQ), and hydroxychloroquine (HCQ) can be initiated as a monotherapy or a combination therapy in the first-line treatment. Combination therapy with two or more csDMARDs may be effective when single-DMARD treatments are unsuccessful or in RA patients presenting with several prognostic factors, such as elevated levels of inflammatory markers (e.g., Erythrocyte Sedimentation Rate [ESR] and C-reactive protein [CRP]), multiple swollen joints, and extra-articular manifestations. The treatment response and dose titration are typically assessed every 1–3 months using multiple criteria, such as ESR and CRP levels, joint pain and swelling, and evaluations from both the patient and physician. Treatment switching usually occurs at 6-month intervals after the initial treatment failure [5, 6]. For those patients exhibiting RA with inadequate response, a series of csDMARDs was sequentially added, namely methotrexate (MTX) + sulfasalazine (SSZ) + leflunomide (LEF) [5]. In the last decade, new biologics (bDMARDs) and their biosimilars (bsDMARDs) as well as targeted synthetic DMARDs (tsDMARDs) for the treatment of RA have been licensed because of their clear clinical benefits in terms of ameliorated disease severity and improved chances for remission [7, 8], albeit at greater costs than the standard treatment [7–9]. In Thailand, when RA patients fail three csDMARDs or experience csDMARD toxicity, only rituximab is reimbursed and then only through the Civil Service Medical Benefit Scheme (CSMBS), one of three available healthcare schemes, via a special program, namely the Rheumatic Disease Prior Authorization (RDPA) [5, 6]. Patients with RA, who are insured under the universal health coverage scheme (UCS) and the social security scheme (SSS) that cover around 90% of the population, can typically access only csDMARDs (such as cyclosporine and azathioprine). In 2019, the National List of Essential Medicines (NLEM) subcommittee, which oversees the Thai pharmaceutical reimbursement list for these three healthcare schemes, required evidence of cost-effectiveness before determining whether bDMARDs, tsDMARDs or bsDMARDs should be included in the essential list as treatments for patients exhibiting RA with high disease activity. This study aims to evaluate the cost-utility of bDMARDs, tsDMARDs and bsDMARDs combined with MTX in RA patients who have had an inadequate response to the typical combination of three csDMARDs (i.e., MTX, SSZ, and LEF) in the Thai context.

## Methods

The costs and health outcomes for the novel treatments were compared with those of the conventional treatment using a model-based economic evaluation. The modelled population consists of RA patients with inadequate responses to the standard combination of three csDMARDs, namely methotrexate (MTX) + sulfasalazine (SSZ) + leflunomide (LEF). Treatment options consisted of the current treatment (i.e., csDMARDs) and one of the seven patented bDMARDs or tsDMARDs, which can be divided into four main groups: 1) anti-TNF-α, i.e., etanercept, infliximab, and golimumab; 2) anti-IL6, i.e., tocilizumab; 3) anti-CD20, i.e., rituximab; and 4) JAK inhibitor, i.e., tofacitinib and baricitinib (Table 1). In addition, the following bsDMARDs that have been approved by the Thai Food and Drug Administration (FDA) were also included as treatment options: biosimilars to infliximab (Remsima® and Ixifi®), a biosimilar to the TNF-α inhibitor adalimumab (Amgevita®), and a biosimilar to rituximab (Truxima®). As recommended by the Thai Health Technology Assessment (HTA) Guideline [10], the analysis was conducted from a societal perspective to estimate lifelong costs and health outcomes (i.e., quality-adjusted life years or QALYs). Both estimated costs and QALYs were discounted by 3% per year. All costs were adjusted for the year 2022 using the consumer price index (CPI) [11]. The cost-effectiveness ceiling threshold of 160,000 THB per QALY gained (US $4,634, 1 USD = 34.53 THB in 2022) was used as recommended by the Health Economic Working Group under the Subcommittee for the Development of the National List of Essential Medicine (NLEM) [12].

**Table 1:** Alternative treatments combined with methotrexate (MTX) for rheumatoid arthritis patients with high disease activity and inadequate responses to three conventional disease-modifying antirheumatic drugs (DMARDs)

Alternative treatments	Generic name (trade name)	Abbreviations used in this study	Route of administration	Unit dose	Dosing regimen
**1**	**Current treatment (Comparator)**	SoC			
**1.1**	Cyclosporine	CsA	Oral	100 mg/cap	200 mg/day
**1.2**	Azathioprine	AZA	Oral	50 mg/tab	100 mg/day
	**New treatments (Intervention)**				
**2**	Etanercept (Enbrel®)	ETA	Subcutaneous injection	25 mg/0.5 ml	25 mg twice weekly
**3**	Infliximab (Remicade®)	IFX	Intravenous infusion	100 mg/vial	3 mg/kg in 250 mg of NSS, IV infusion at least 2 h at 0, 2, and 6 weeks, followed by a maintenance regimen of 3 mg/kg every 8 weeks thereafter
**4**	Golimumab (Simponi®)	GOL	Subcutaneous injection	50 mg/0.5 ml	50 mg once a month
**5**	Tocilizumab (Actemra®)	TCZ	Subcutaneous injection	162 mg/0.9 ml	162 mg (< 100 kg) every two weeks
**6**	Rituximab (Mabthera®)	RTX	Intravenous infusion	500 mg/50 ml	1,000 mg on days 1 and 15 every 6 months
**7**	Tofacitinib (Xeljanz®)	TOF	Oral	5 mg/tab	5 mg BID
**8**	Baricitinib (Olumiant®)	BAR	Oral	4 mg/tab	4 mg OD
**9**	Biosimilar infliximab (Remsima®)	bsIFXr	Intravenous infusion	100 mg/vial	3 mg/kg in 250 mg of NSS, IV infusion at least 2 h at 0, 2, and 6 weeks, followed by a maintenance regimen of 3 mg/kg every 8 weeks thereafter
**10**	Biosimilar infliximab (Ixifi®)	bsIFXi	Intravenous infusion	100 mg/vial	3 mg/kg in 250 mg of NSS, IV infusion at least 2 h at 0, 2, and 6 weeks, followed by a maintenance regimen of 3 mg/kg every 8 weeks thereafter
**11**	Biosimilar Adalimumab (Amgevita®)	bsADA	Subcutaneous injection	40 mg/0.8 ml	40 mg biweekly
**12**	Biosimilar Rituximab (Truxima®)	bsRTX	Intravenous infusion	500 mg/50 ml	1,000 mg on days 1 and 15 every 6 months

*mg* milligram, *cap* capsule, *kg* kilogram, *ml* milliliter, *tab* tablet, *IV* Intravenous infusion, *NSS* Normal saline solution

## Study overview

**Fig. 1:**
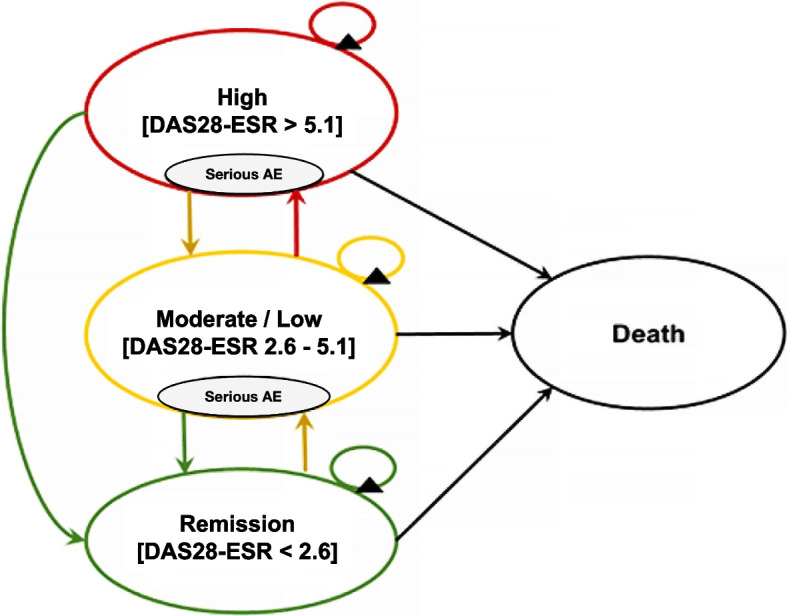
Markov model: Model structure represents the disease progression of rheumatoid arthritis patients with high disease activity measured by DAS28-ESR. DAS-28: Disease Activity Score with 28 joint counts, ESR: erythrocyte sedimentation rate

A Markov model was constructed using Microsoft Excel (Microsoft Corp., Redmond, Washington, USA) to simulate disease progression. Four health states were classified according to the Disease Activity Score-28 for RA with ESR (DAS28-ESR) criteria [13–16], consisting of 1) high disease activity, defined as DAS28-ESR > 5.1; 2) moderate or low disease activity, defined as DAS28-ESR ≥ 2.6 to 5.1; 3) remission defined as DAS28-ESR < 2.6; and 4) death (Fig. 1). The model was constructed with a cycle length of 6 months to measure treatment response using DAS28-ESR criteria [6]. The cohort population of the analysis was composed of 56-year-old RA patients with high disease activity (DAS28-ESR > 5.1) who had an inadequate response to three csDMARDs. The second-treatment sequence for RA patients with inadequate response was implemented as follows: 1) Those on the novel treatment + MTX regimen would be switched to the SoC + MTX treatment; and 2) Those already on the SoC + MTX regimen would remain on this treatment course. Supportive care, involving the use of corticosteroids or non-steroidal anti-inflammatory drugs (NSAIDs), supplemented both treatment arms, resulting in an SoC + MTX + supportive care regimen for all patients with inadequate response (Additional file 1). To manage RA patients in remission for 6–12 months, the Thai clinical practice guideline recommends several strategies, including dose tapering, increasing dosing intervals, or discontinuing bDMARDs, tsDMARDs or bsDMARDs [6]. In this study, RA patients who achieved remission would receive treatment for six months before discontinuing all treatments except for MTX, which would continue unchanged. Patients who experienced a serious adverse event (SAE) discontinued their current regimen for 6 months while they received treatment for their SAE before the resumption of RA treatment. The model assumptions were validated and approved by experts and stakeholders during a consultation meeting according to the second edition of the Thai HTA Guideline [10] and the Thai HTA process guideline [17].

## Model inputs

### Transitional probabilities

#### Disease progression, response to standard of care, and mortality

For current treatments, the transitional probabilities, describing 6-month increments of changing RA disease activity based on DAS28-ESR values (Fig. 1), were calculated from a survival analysis using 5-year retrospective clinical data (from 2015 to 2019) in three tertiary hospitals (Table 2). A total of 84 RA patients were reviewed, each treated with the standard of care (SoC) after inadequate response to three csDMARDs. The mean age of the patients was 55.56 years (SD 13.36), with mean DAS28-ESR of 4.85 (SD 1.25) and median DAS28-ESR of 4.82 (IQR 4.13–5.57). The patients were primarily women (92%) (Additional file 2). Data from the reviewed patients were examined according to events of interest as follows: 1) Transition from high disease activity to moderate / low disease activity, 44 events; 2) Transition from high disease activity to remission, 12 events; 3) Transition from moderate / low disease activity to high disease activity, 23 events; 4) Transition from moderate / low disease activity to remission, 18 events; and 5) Transition from remission to moderate / low disease activity, 12 events. A parametric survival-time model with the Weibull distribution was applied to derive time-dependent probabilities for each health state transition using STATA (StataCorp. 2019. Stata Statistical Software: Release 16. College Station, TX: StataCorp LLC.). The survival function, S(t), is [18]:

$$S\left(t\right) =exp\left\{-H\left(t\right)\right\}$$

along with

$$H\left(t\right) = {\lambda t}^{\gamma }$$

$$tp\left(u\right) =1-exp\left\{\lambda {\left(t-u\right)}^{\gamma } - {\lambda t}^{\gamma }\right\}$$

*where S(t)* = probability of survival as a function of time; *H(t)* = cumulative hazard function of the Weibull distribution; *λ* (lambda) = scale parameter; *t* = time in 6-month periods; *γ* (gamma) = shape parameter; *tp(u)* = transitional probability of an event during the cycle, and *u* = cycle length of the model.

**Table 2:** Model input parameters

Input parameters	Mean value (standard error)	Distribution for probabilistic analyses	Source
**1. Relative risk of death in patients with RA who had high disease activity**	2.43 (1.22)	Lognormal	[3]
**2. Probability of adverse events**^**c**^	0.0008 (0.00003)	Beta	^a^
**3. Survival analysis**			
** 3.1 Transition from high to moderate / low disease activity**			
Constant in survival analysis for baseline hazard	1.55	Lognormal	Medical record review
Disease duration coefficient in survival analysis for baseline hazard	-0.08	Lognormal	
Lambda parameter survival analysis (depends on chosen coefficients)	1.61	Lognormal	
Ancillary (shape) parameter in the Weibull distribution	1.08	Lognormal	
Mean disease duration of RA patients from 3 hospitals	14	Lognormal	
** 3.2 Transition from high disease activity to remission**			
Constant in survival analysis for baseline hazard	-2.92	Lognormal	Medical record review
Lambda parameter survival analysis (depends on chosen coefficients)	0.05	Lognormal	
Ancillary (shape) parameter in the Weibull distribution	1.00	Lognormal	
** 3.3 Transition from moderate / low to high disease activity**			
Constant in survival analysis for baseline hazard	-1.39	Lognormal	Medical record review
Lambda parameter survival analysis (depends on chosen coefficients)	0.25	Lognormal	
Ancilliary (shape) parameter in Weibull distribution	0.50	Lognormal	
** 3.4 Transition from moderate / low disease activity to remission**			
Constant in survival analysis for baseline hazard	-1.86	Lognormal	Medical record review
Lambda parameter survival analysis (depends on chosen coefficients)	0.16	Lognormal	
Ancillary (shape) parameter in the Weibull distribution	0.63	Lognormal	
** 3.5 Transition from remission to moderate / low disease activity**			
Constant in survival analysis for baseline hazard	-0.22	Lognormal	Medical record review
Lambda parameter survival analysis (depends on chosen coefficients)	0.80	Lognormal	
Ancillary (shape) parameter in the Weibull distribution	0.96	Lognormal	
**4. Costs (adjusted to 2022 value)**			
** 4.1 Direct medical costs (THB)**			
OPD service (per visit)	78	-	[19]
IV infusion (per visit)	31	-	
Treatment for serious adverse events (per admission)^c^	36070 (1274)	Gamma	^a^
Drug costs (THB per 6 months)			
Standard of care + MTX	18700	-	Reference drug price from DMSIC
Etanercept + MTX	108500	-	^b^
Infliximab + MTX	168700	-	^b^
Golimumab + MTX	217400	-	^b^
Tocilizumab + MTX	102400	-	^b^
Rituximab + MTX	98000	-	^b^
Tofacitinib + MTX	89000	-	^b^
Baricitinib + MTX	89200	-	^b^
Biosimilar infliximab (Remsima®) + MTX	101500	-	^b^
Biosimilar infliximab (Ixifi®) + MTX	68000	-	^b^
Biosimilar adalimumab (Amgivita®) + MTX	90300	-	^b^
Biosimilar rituximab (Truxima®) + MTX	78600	-	^b^
Lab costs before starting treatment (THB at the first visit for every regimen)			
X-ray (Hand)	265.04	-	[19]
X-ray (Foot)	531.25	-	
X-ray (Chest)	265.04	-	
Complete blood count (CBC)	140.05	-	
Erythrocyte sedimentation rate (ESR)	62.50	-	
Test for TB (modified AFB)	108.80	-	
Liver function test (LFT)	546.29	-	
Serum creatinine (SCr)	77.55	-	
Lipid profile	312.50	-	
** 4.2 Direct non-medical cost (THB per visit)**			
Transportation fare	329.97	-	[19]
Food	121.55	-	
**5. Number of OPD visits (time per 6 months)**			
High disease activity	6	-	[5]
Moderate / Low disease activity	2	-	
Remission	1	-	
**6. Utilities**			
High disease activity	0.79 (0.05)	Beta	[20]
Moderate / Low disease activity	0.86 (0.01)	Beta	
Remission	0.93 (0.01)	Beta	

*MTX* Methotrexate, *DMSIC* Drug and Medical Supply Information Center

^a^Calculated from the national database (National Health Security Office: NHSO)

^b^Drug price submitted by pharmaceutical manufacturers

^c^Serious AE of interest included serious infection, tuberculosis, herpes zoster, thromboembolism, cancer, and cardiovascular events

**Fig. 2:**
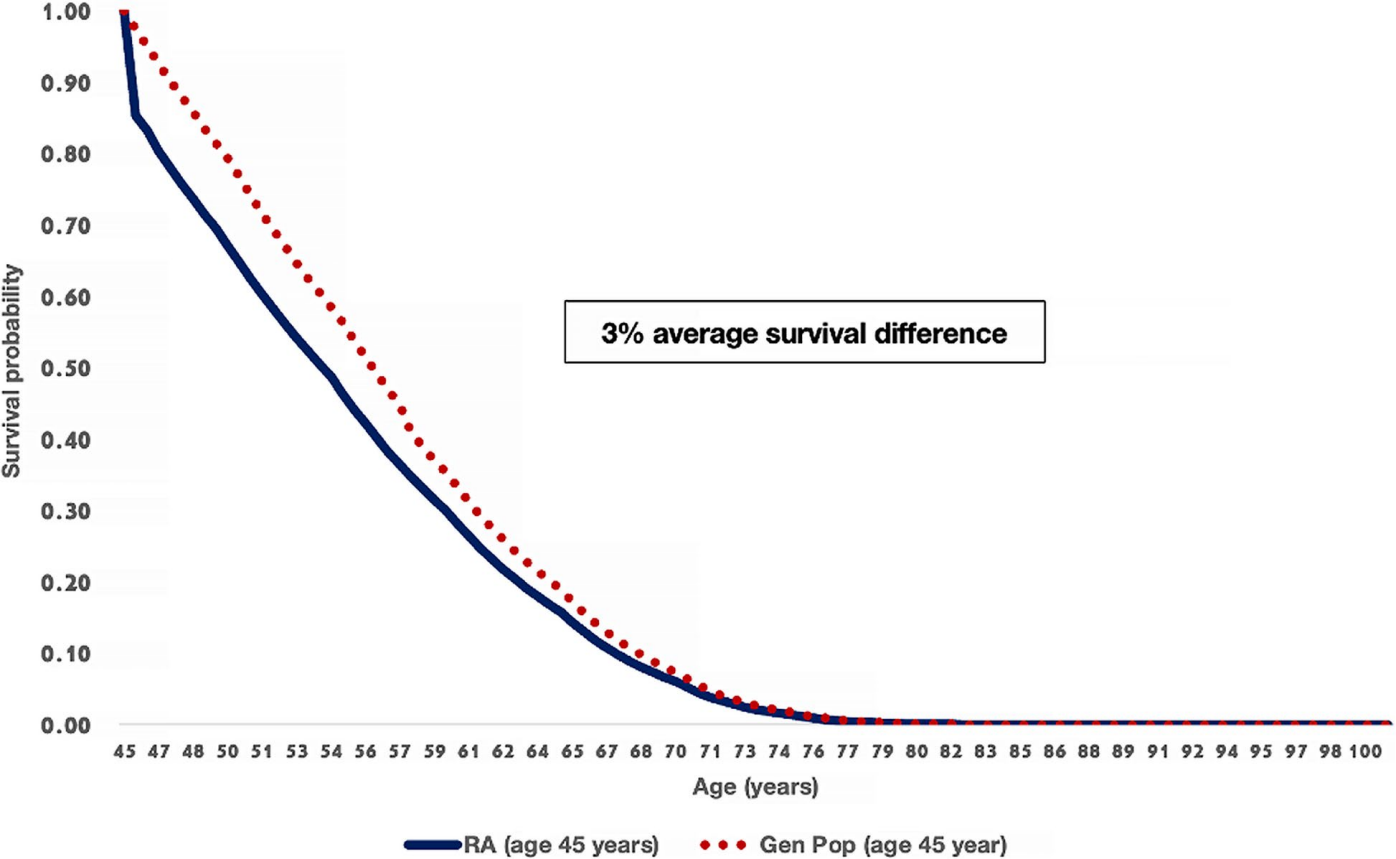
Model validation: Survival curves estimated from an analytical model comparing RA patients aged 45 years at diagnosis with the general population aged 45 years

The probabilities of death in RA with moderate / low disease activity and remission were obtained from the Thai life table [21], with the provision that the probability of death in patients with high disease activity was adjusted by a hazard ratio of 2.43 (95% CI 1.64 to 3.61) derived from an observational study in Germany [3]. Figure 2 shows the predicted survival curve for RA patients with inadequate response to 3 csDMARDs who received the SoC compared to the general population. RA patients had a poorer survival rate than the general population by 3%.

#### Efficacy and safety of bDMARDs, tsDMARDs, and bsDMARDs

A systematic review and network meta-analysis (SR-NMA) were used to assess the treatment effects and safety of bDMARDs, tsDMARDs, and bsDMARDs at 6, 12, and 24 months. The full details of this SR-NMA were reported previously [22]. In short, electronic database searches were performed in Medline / PubMed, EMBASE, and the Cochrane Library from inception until November 30, 2021, without language restrictions. Database search strategies were adapted from a previously published Cochrane review [7]. The reference list of all eligible studies was reviewed to ensure that no relevant studies were missed. The title or abstract and full text articles that met the predefined inclusion criteria (Additional file 3) were independently screened and extracted by four review authors (NB, JP, SP, CC). A total of 44 unique articles were included in the network meta-analysis and grouped by the outcome of interest measured at 6 and 12 months as follows: 1) Clinical remission or DAS28-ESR < 2.6 (transition from high disease activity or DAS28-ESR > 5.1 to remission, “High to Remission”) identified 41 studies for 6 months and 18 studies for 12 months; 2) Transition from clinical high disease activity to moderate / low disease activity (DAS28-ESR > 2.6 to 5.1**,** “High to Moderate / low”) identified 9 studies for 6 months and 10 studies for 12 months; and 3) Instances of serious infection identified 33 studies [22]. Serious infection was selected to represent a serious adverse event based on suggestions from medical experts. We performed the network meta-analysis within a frequentist framework using multivariate meta-analysis estimated by restricted maximum likelihood [23]. All analyses were performed with STATA (StataCorp. 2019. Stata Statistical Software: Release 16. College Station, TX: StataCorp LLC.). The findings are summarized in terms of relative risk (RR) with 95% confidence intervals for each treatment option compared to MTX monotherapy. The main findings of the SR-NMA are described in Additional file 4.1.

In the absence of some efficacy data, the Markov model was used with the following efficacy assumptions, which were agreed upon during the stakeholder meeting: 1) For the efficacy of “High to Remission,” if the outcome of interest from eligible trials was unavailable at some time points, we assumed the same efficacy at every time point of the outcome measured; and 2) Because there was no evidence for the proportion of clinical patients with moderate / low disease activity who achieved remission, this efficacy was assumed to be comparable to the state of “High to Remission” (Additional file 4.2).

#### Cost parameters

The societal perspective was used to estimate the lifetime costs of RA patients. All costs were reported in year 2022 values. Direct medical and non-medical care costs incurred by each treatment option were considered (Table 2). Indirect costs were excluded from this analysis according to the double counting issue suggested by the Thai HTA guideline [10]. All treatment-related costs were estimated for each health state, with costs for the first six months partitioned from those of all subsequent months. Direct medical care costs included medication costs, cost of pretreatment screenings (i.e., chest radiographs and laboratory tests), treatment of serious adverse events, drug administration fees (only infliximab, rituximab and their biosimilars), laboratory tests for safety monitoring and outpatient fees. To estimate the aforementioned costs, the guideline for Thai RA diagnosis and treatment as well as the Thai standard costing menu were referenced [5, 6, 19] to account for resources used. The costs of bDMARDs and tsDMARDs were based on the prices announced by the Ministry of Public Health Drug and Medical Supply Information Center (DMSIC) [24] and the prices submitted by pharmaceutical companies for this study. The costs of treating serious adverse events including serious infection, tuberculosis, herpes zoster, thromboembolism, cancer, and cardiovascular events were obtained from the Central Office of Healthcare Information and the National Health Security Office (NHSO) databases. Direct non-medical care costs covered accommodation, travel, food costs for patients and caregivers, and opportunity costs incurred by caregivers. These costs were estimated based on the Thai standard costing menu and the frequency of hospital visits [19].

#### Utility values

The utility of Thai patients with RA was obtained from a previously published study [20]. Briefly, the Thai version of the EuroQol Quality of Life in five dimensions five level version (EQ-5D-5L) questionnaire was used to interview 464 patients from the Rheumatoid Arthritis registries of the Siriraj and Phramongkutklao university hospitals with a mean age of 59.15 years and a mean duration of the disease of 11.53 years. The utility values were reanalyzed by WK to align with the DAS28-ESR health states used in this study (Table 2).

#### Uncertainty analysis

To determine parameter uncertainty and the robustness of the results, one-way and probabilistic sensitivity analyses were performed. For one-way sensitivity analysis, key parameters such as efficacy of novel treatments, utility values, costs, and mortality rate were examined to see how changes in these parameters affected incremental cost-effectiveness ratios (ICERs) in the base-case scenario. To conduct the probabilistic sensitivity analysis (PSA) a second-order Monte Carlo simulation was performed 5,000 times in Microsoft Excel (Microsoft Corp., Redmond, Washington, USA), yielding a range of plausible values for lifetime costs, QALYs, and ICERs. The model parameters were randomly sampled based on mean, standard error (SE), and distribution of each parameter as follows [25]: 1) beta distribution, for transitional probabilities and utility scores because the values ranged from 0 to 1; 2) gamma distribution, for cost variables because these parameters must be positive values; and 3) log normal distribution, for survival parameters.

## Results

**Fig. 3:**
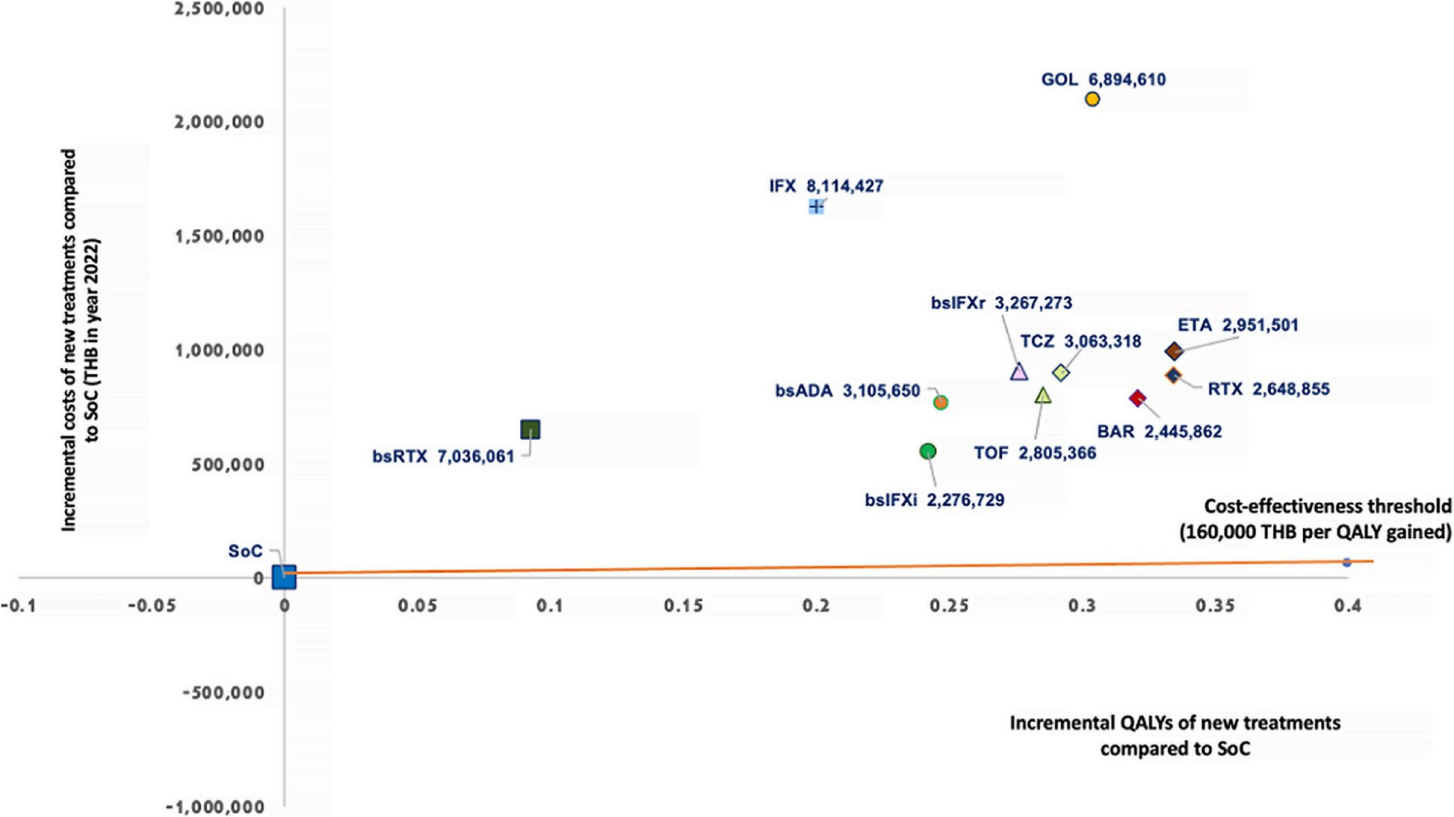
Cost-effectiveness plane: A comparison of incremental costs, incremental QALYs, and ICERs between the standard of care (SoC) and new treatments using bDMARDs, tsDMARDS, and bsDMARDs in combination with MTX. QALYs: quality-adjusted life years, ICERs: incremental cost-effectiveness ratios, bDMARD: biologic disease-modifying antirheumatic drug, tsDMARD: targeted synthetic disease-modifying antirheumatic drug, SoC: Standard of care, MTX: Methotrexate, ETA: Etanercept (Enbrel®), IFX: Infliximab (Remicade®), GOL: Golimumab (Simponi®), TCZ: Tocilizumab (Actemra®), RTX: Rituximab (Mabthera®), TOF: Tofacitinib (Xeljanz®), BAR: Baricitinib (Olumiant®), bsIFXr: Biosimilar infliximab (Remsima®), bsIFXi: Biosimilar infliximab (Ixifi®), bsADA: Biosimilar adalimumab (Amgevita®), bsRTX: Biosimilar rituximab (Truxima®)

Lifetime costs, QALYs, and ICERs are shown in Table 3. In combination with MTX, the lifetime cost of novel treatments ranged from 0.8 to 2.3 million THB (US $23,102 to $65,935). Golimumab had the highest lifetime cost of 2.3 million THB (US $65,935), followed by infliximab at 1.9 million THB (US $54,202), and etanercept at 1.2 million THB (US $35,771). The remaining novel treatments had lifetime costs ranging from 0.8 to 1.1 million THB (US $23,102 to $25,990). In contrast, the SoC had the lowest lifetime cost at 0.2 million THB (US $7,145). Life year (LY) and QALY values, under the discounted annual rate, were comparable between the novel treatments and the SoC (LYs of 5.70–5.97 vs. 5.61 and QALYs of 4.90–5.14 vs. 4.80 for the new treatments versus the SoC, respectively). Compared to the SoC, all new treatments had substantially higher costs with only slightly improved QALYs. In the Thai setting, none of the new treatments were cost-effective compared to the ceiling threshold of 160,000 THB (US $4,634) per QALY gained [12]. The ICER values of the new treatments ranged from 2.3 to 8.1 million THB (US $65,935 to $234,996) per QALY gained, with the biosimilar infliximab having the lowest ICER value at 2.3 million THB (US $65,935) per QALY gained (Fig. 3).

**Table 3:** Lifetime cost, life year (LY), quality-adjusted life year (QALY) and incremental cost-effectiveness ratio (ICER): Base-case analysis (patients aged 56 years)

**Base-case**^a^	**Treatment options**^c^											
	**SoC**	**ETA**	**IFX**	**GOL**	**TCZ**	**RTX**	**TOF**	**BAR**	**bsIFXr**	**bsIFXi**	**bsADA**	**bsRTX**
Discounted lifetime costs^b^	246728	1235188	1871581	2343472	1141704	1132732	1047153	1032029	1149523	797714	1013749	897446
Life year	6.52	6.94	6.77	6.87	6.87	6.93	6.91	6.93	6.84	6.81	6.82	6.62
Discounted Life years^b^	5.61	5.97	5.82	5.91	5.90	5.96	5.94	5.96	5.88	5.85	5.86	5.70
QALYs	5.59	5.98	5.83	5.95	5.94	5.99	5.92	5.97	5.92	5.88	5.88	5.70
Discounted QALYs^b^	4.80	5.14	5.00	5.11	5.10	5.14	5.09	5.12	5.08	5.05	5.05	4.90
Incremental costs^b^		988460	1624853	2096744	894976	886004	800425	785300	902795	550986	767021	650718
Incremental life years^b^		0.36	0.21	0.30	0.29	0.35	0.33	0.35	0.27	0.24	0.25	0.09
Incremental QALYs^b^		0.33	0.20	0.30	0.29	0.33	0.29	0.32	0.28	0.24	0.25	0.09
ICER (THB/QALY gained)^b^		2951501	8114427	6894610	3063318	2648855	2805366	2445862	3267273	2276729	3105650	7036061

*SoC* Standard of care, *bs* biosimilar, *ETA* Etanercept (Enbrel®), *IFX* Infliximab (Remicade®), *GOL* Golimumab (Simponi®), *TCZ* Tocilizumab (Actemra®), *RTX* Rituximab (Mabthera®), *TOF* Tofacitinib (Xeljanz®), *BAR* Baricitinib (Olumiant®), *bsIFXr* Biosimilar infliximab (Remsima®), *bsIFXi* Biosimilar infliximab (Ixifi®), *bsADA* Biosimilar adalimumab (Amgevita®), *bsRTX* Biosimilar rituximab (Truxima®)

^a^The average values were obtained using a probabilistic model with 5,000 iterations

^b^Discounted by 3% annually

^c^All treatments were combined with methotrexate

### Uncertainty analyses

**Fig. 4:**
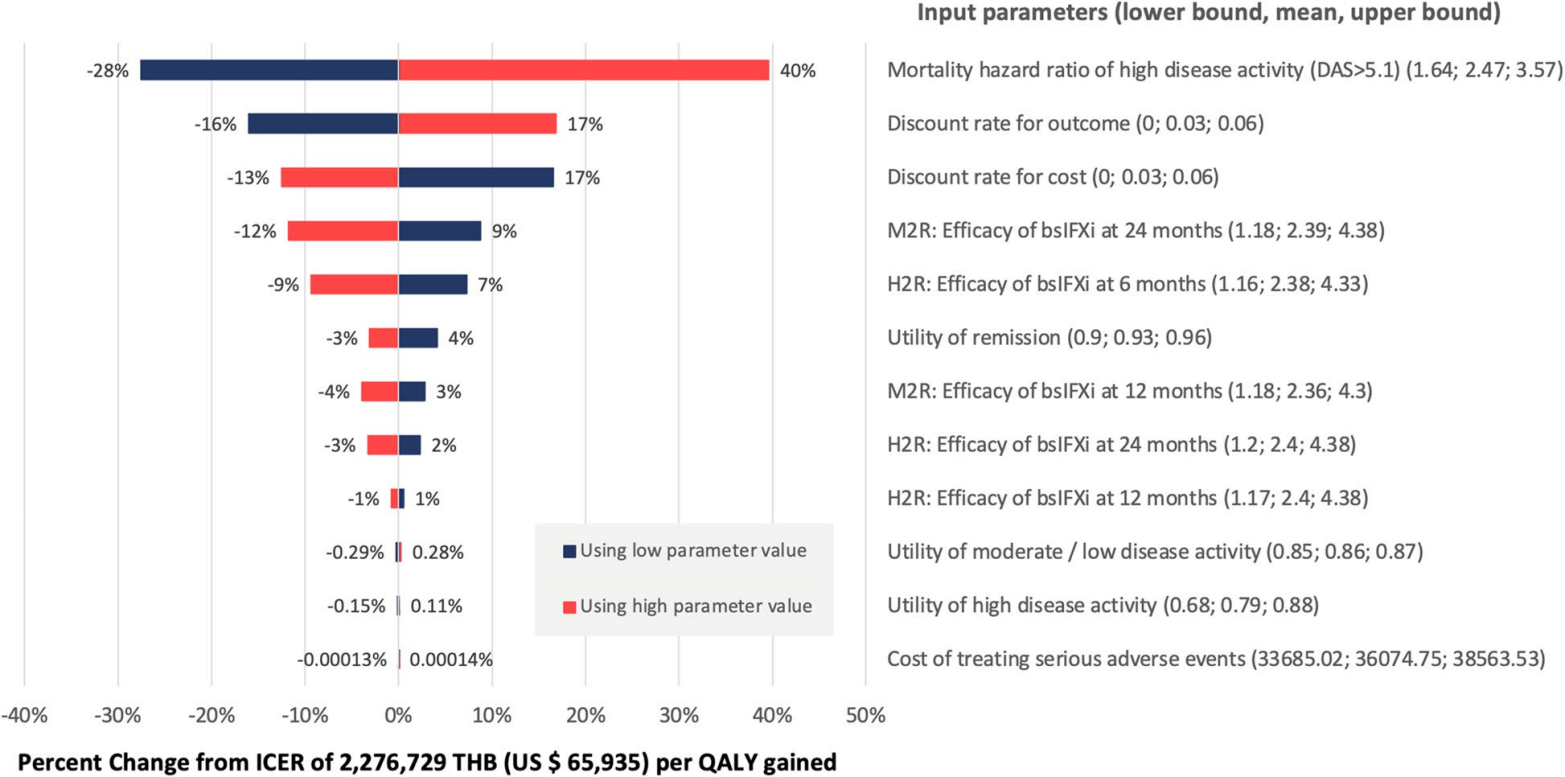
One-way sensitivity analysis: Tornado diagram indicates the percentage of change from mean ICER when each parameter is varied in its plausible range. Only the biosimilar infliximab (Ixifi®) + methotrexate result was selected for analysis. DAS: Disease Activity Score, M2R: transitional probability from moderate / low disease activity to remission, H2R: transitional probability from high disease activity to remission, bsIFXi: Biosimilar infliximab (Ixifi®)

**Fig. 5:**
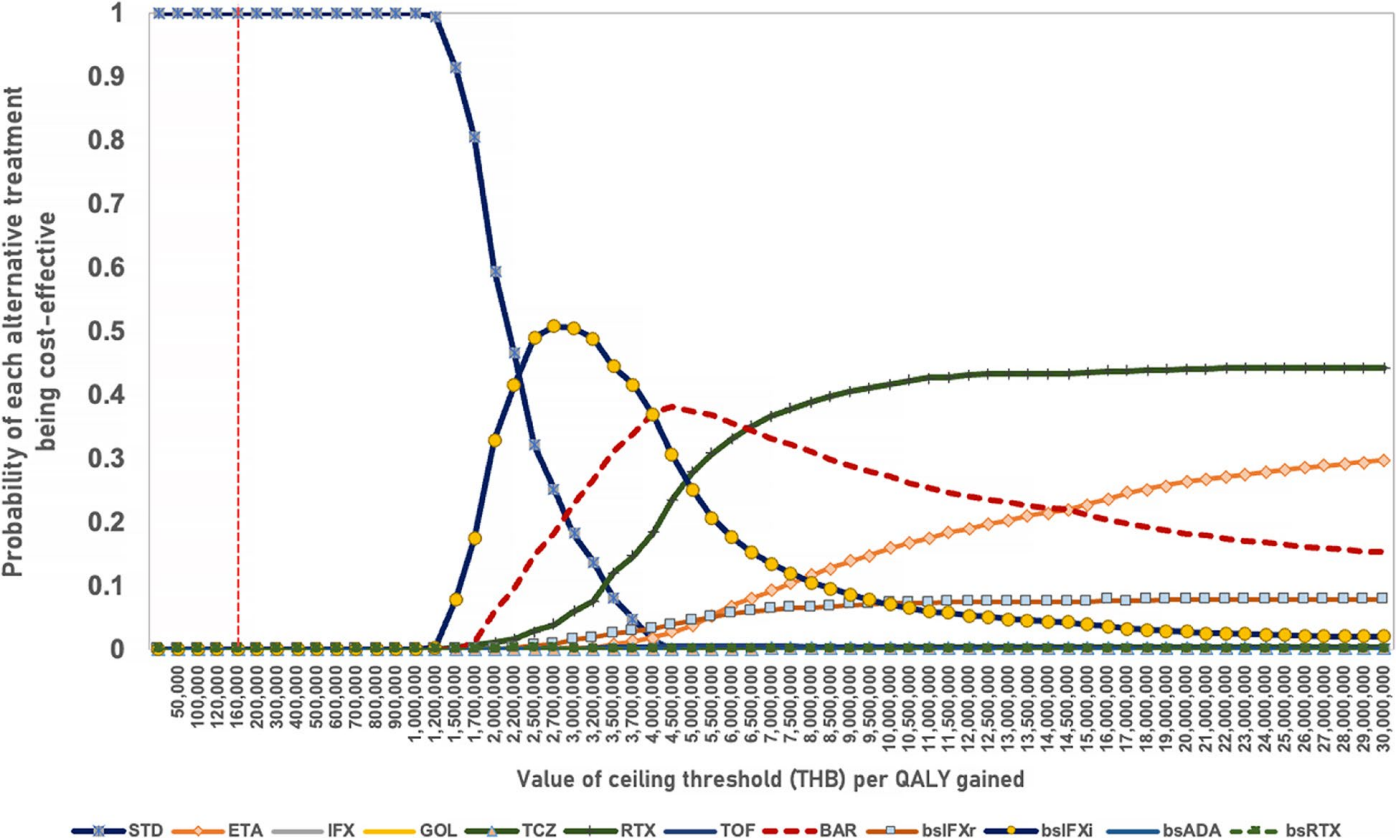
Cost-effectiveness acceptability curve of probabilistic sensitivity analysis: This graph presents the probability of being the optimal option at a given cost-effectiveness threshold compared to all alternative treatments in combination with methotrexate. The dashed line represents the ceiling threshold for cost-effective health technology in Thailand. ETA: Etanercept (Enbrel®), IFX: Infliximab (Remicade®), GOL: Golimumab (Simponi®), TCZ: Tocilizumab (Actemra®), RTX: Rituximab (Mabthera®), TOF: Tofacitinib (Xeljanz®), BAR: Baricitinib (Olumiant®), bsIFXr: Biosimilar infliximab (Remsima®), bsIFXi: Biosimilar infliximab (Ixifi®), bsADA: Biosimilar adalimumab (Amgevita®), bsRTX: Biosimilar rituximab (Truxima®)

Results from the one-way sensitivity analysis are depicted in the tornado diagram (Fig. 4). The one-way sensitivity analysis showed that the mortality hazard ratio of high disease activity was the input parameter with the greatest influence. The PSA results were presented as a cost-effectiveness acceptability curve (CEAC). The PSA, consisting of 5,000 simulations, demonstrated that no new alternative treatments had a chance of being cost-effective at the ceiling threshold of 160,000 THB (US $4,634) per QALY gained. The biosimilar of infliximab (Ixifi®), baricitinib, rituximab, etanercept and the biosimilar of infliximab (Remsima®) were more likely to be cost-effective if the ceiling threshold were set higher than 2.5 million THB (US $72,401) per QALY gained, whereas the other treatments were prohibitively expensive. When the threshold was 3 million THB (US $86,881) per QALY gained, the probability of being cost-effective for the biosimilar of infliximab (Ixifi®) reached its peak at 50% (Fig. 5).

## Discussion

Given a national ceiling threshold of 160,000 THB (US $4,634) per QALY gained, our results show that neither bDMARDs, tsDMARDs nor bsDMARDs combined with MTX treatment for eligible RA patients were cost-effective in the Thai setting [12]. The sensitivity analysis confirms the robustness of the estimated results. In addition, uncertainty in treatment prices, any of which can result in higher costs than conventional treatments using csDMARDs from the current health coverage reimbursement list, had no effect on the cost-effectiveness.

In this multicenter study, data on clinical outcomes (DAS28-ESR values) for the current treatment regimen were collected from three centers and aligned with the evaluation of a patient's outcome in clinical practice [5], making it relevant to the local context. In addition, the quality of life related to health among Thai patients with RA was directly collected using the EQ-5D questionnaire and reanalyzed to conform to the analytical model used in this study [17, 20]. Furthermore, following the recommendation of the national guidelines for the evaluation of health technologies [10, 26], this study examined all available treatments involving bDMARDs, tsDMARDs, and bsDMARDs that were licensed and approved by the Thai FDA for patients with RA and compared all costs and health outcomes, making it the first economic evaluation of this kind in Thailand. This study also used a systematic review and network meta-analysis (SR-NMA) to estimate the efficacy of each treatment option, as opposed to prior economic evaluation studies [27] that assessed cost-utility based on the “generic name” of biologic DMARDs and relied on efficacy data from randomized clinical trials (RCTs) of the original product, with the assumption that treatment outcomes for biosimilar DMARDs and their original products were equivalent.

Our findings are consistent with those of previous studies in Sweden [28], Iran [29], the Netherlands [30] and the United Kingdom [31] indicating that bDMARDs or tsDMARDs were not cost-effective across many contexts due to their high cost. However, previous studies were conducted in patients with RA with moderate disease activity, where bDMARDs or tsDMARDs were used when MTX therapy failed, whereas our RA patients had more severe disease activity and failed multiple lines of treatment, i.e., inadequately responded to three csDMARDs. It has been established that disease progression, as a consequence of consecutive treatment failures and the delayed addition of bDMARDs / tsDMARDs to a methotrexate regimen, leads to reduced efficacy rates and adverse health outcomes [32]. As a result, the prior studies reported better health outcomes owing to the earlier use of bDMARDs / tsDMARDs in the treatment of eligible patients. Furthermore, bDMARDs and tsDMARDs could be relatively cost effective compared to one another, but they were never cost effective overall when compared to the SoC. For example, adalimumab was found to be cost-effective compared to the TNF antagonist that was included in the national list of essential medicines in Sweden [28].

This analysis has certain limitations. First, the response rates of the American College of Rheumatology (ACR) are widely used as a primary outcome to measure the efficacy of bDMARDs, tsDMARDs, and their bsDMARDs. However, the studies with ACR results were excluded from our SR-NMA because the present study used DAS28-ESR values as the outcome of interest, as suggested by the clinical practice guidelines [5]. Second, because of limited resources, the RA treatment sequence recommended by the Thai RA treatment guidelines is not aligned with the ACR 2015 / EULAR 2016 guidelines [33, 34]. Therefore, the efficacies of bDMARDs, tsDMARDs, and their bsDMARDs obtained from the SR-NMA were only compared with that of MTX, whereas the SoC in this study was cyclosporine or azathioprine combined with MTX. Third, although there were efficacy assumptions, they were transparently validated by rheumatologists in stakeholder consultation meetings. These included (1) assuming that treatments without efficacy data in the “transition from high disease activity to remission” would be equivalent to the efficacy values for the same treatments where data was available, and (2) assuming that the efficacy of the “transition from moderate / low disease activity to remission” would be comparable to the “transition from high disease activity to remission.” Fourth, although practitioners using bDMARDs, tsDMARDs, and bsDMARDs reported different serious adverse events (i.e., tuberculosis, herpes zoster, thromboembolism, neoplasm, and serious infection), the study rheumatologist was most concerned with serious infection during treatment. As a result, values for the relative risks of serious infection obtained from the SR-NMA were chosen to represent serious adverse events from the use of bDMARDs, tsDMARDs, and bsDMARDs. Nonetheless, all treatment options were adjusted with the baseline probability of all serious adverse events analyzed from the national health administrative database. Lastly, the risk of death in RA patients with high disease activity was calculated using the mortality hazard ratio of an observational study in Germany, adjusted with the value for the risk of death in the Thai general population. This parameter had an impact on the increase in mortality with age and significantly influenced the incremental cost-effectiveness result, encouraging further research in the Thai RA registry to evaluate mortality classified by disease severity. It can also be applied contextually as real-world evidence for the next HTA research and encourages efficient monitoring and treatment in Thai RA patients with high disease activity.

## Conclusions

The combination of MTX with bDMARDs, tsDMARDs or bsDMARDs for the treatment of RA patients with high disease activity showed a clinically-relevant reduction in disease activity and an increased chance of remission, leading to an improvement in patient quality of life related to health. However, based on the Thai ceiling threshold of 160,000 THB (US $4,634) per QALY, none of these treatment options were cost-effective at their current prices. The findings of this study can be used as evidence for the National List of Essential Medicines (NLEM) subcommittee, comprised of decision makers for the Thai pharmaceutical reimbursement list, to guide its price negotiation process and ensure that these effective drugs are financially affordable before they are added to the NLEM.

## Supplementary Information

**Additional file 1.** Treatment sequence.

**Additional file 2.** Survival analysis.

**Additional file 3.** Inclusion criteria based on the PICOS framework.

**Additional file 4.** Main findings of the SR-NMA.

## Data Availability

The datasets used and / or analysed during the current study are available from the corresponding author upon reasonable request.
