# Supplementary material for: Cost-utility analysis of biologic disease-modifying antirheumatic drugs (bDMARDs), targeted synthetic DMARDs (tsDMARDs) and biosimilar DMARDs (bsDMARDs) combined with methotrexate for Thai rheumatoid arthritis patients with high disease activity
Source: BMC Health Serv Res. 2023 May 31;23:561. doi: 10.1186/s12913-023-09595-1 (PMC10230705; doi:10.1186/s12913-023-09595-1)
Supplement: Supplementary file 1 — Additional file 1. Treatment sequence. [file 12913_2023_9595_MOESM1_ESM.docx]

## Additional file 1. Treatment sequence

### *Treatment sequence for RA patients with inadequate response*


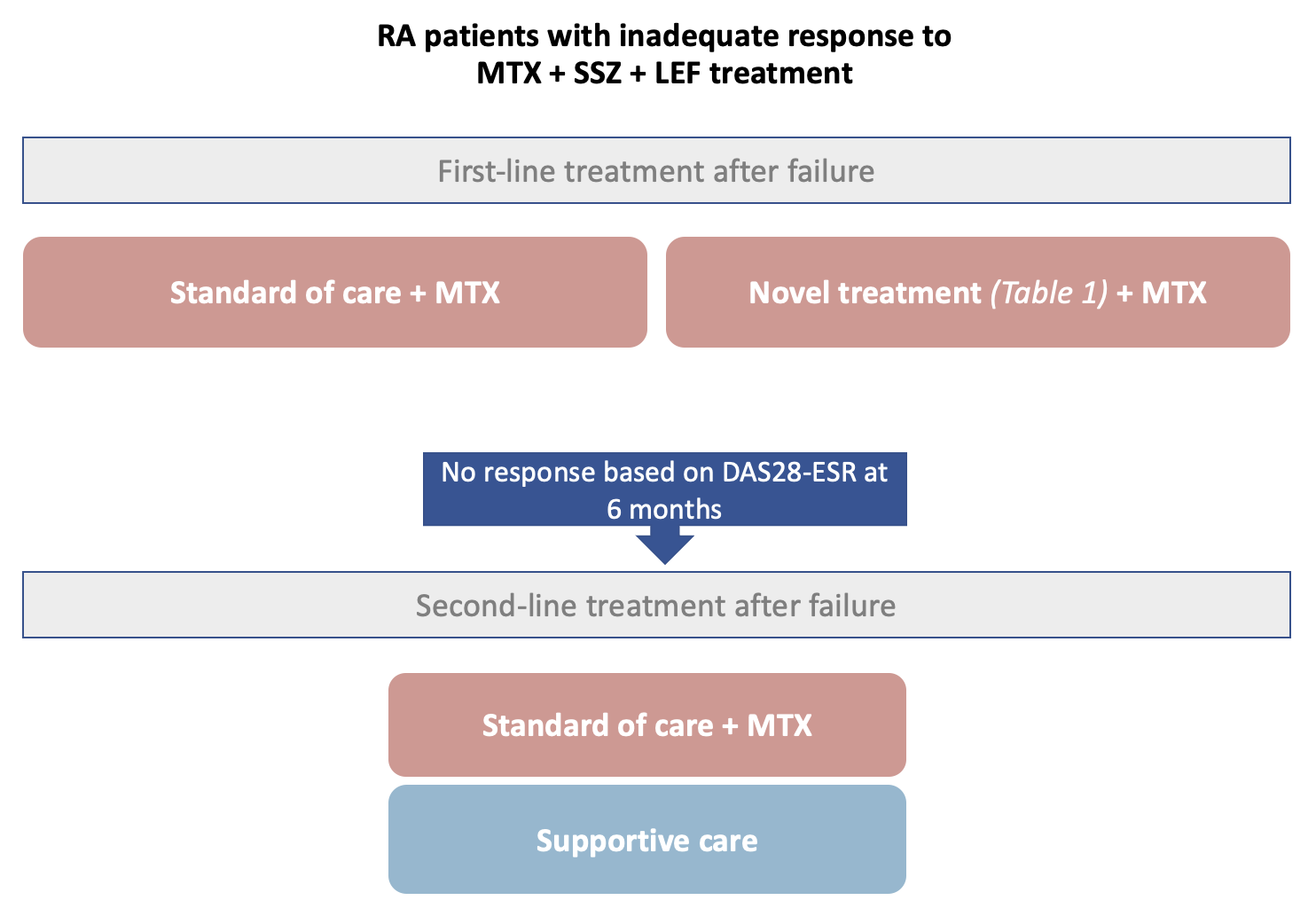


Treatment sequence of RA patients with inadequate response to MTX + SSZ + LEF treatment proceeds as follows:

Intervention arm

**First-line therapy:** Novel treatment + MTX 🡪 **Second-line therapy:** SoC + MTX + Supportive care

Comparator arm

**First-line therapy:** SoC + MTX 🡪 **Second-line therapy:** SoC + MTX + Supportive care

Supportive care includes the use of corticosteroids or non-steroidal anti-inflammatory drugs (NSAIDs).
