# Supplementary material for: Cost-utility analysis of biologic disease-modifying antirheumatic drugs (bDMARDs), targeted synthetic DMARDs (tsDMARDs) and biosimilar DMARDs (bsDMARDs) combined with methotrexate for Thai rheumatoid arthritis patients with high disease activity
Source: BMC Health Serv Res. 2023 May 31;23:561. doi: 10.1186/s12913-023-09595-1 (PMC10230705; doi:10.1186/s12913-023-09595-1)

## Additional file 2. Survival analysis

### *Baseline characteristics of RA patients in 3 tertiary hospitals*

| **Baseline characteristics** | **Number of RA patients (n=84)** |
| --- | --- |
| Sex (male/female) | 7/77 |
| Mean age (SD), year | 60.06 (13.36) |
| Mean age at RA diagnosis (SD), year | 45.63 (12.87) |
| Mean age at inadequate response to combination of 3 csDMARDs (SD), year | 55.56 (55.45) |
| Mean disease duration (SD), year | 14.43 (14.54) |
| Mean DAS28-ESR (SD) | 4.85 (1.25) |
| Median DAS28-ESR (IQR) | 4.82 (4.13-5.57) |

*Results of survival analysis*
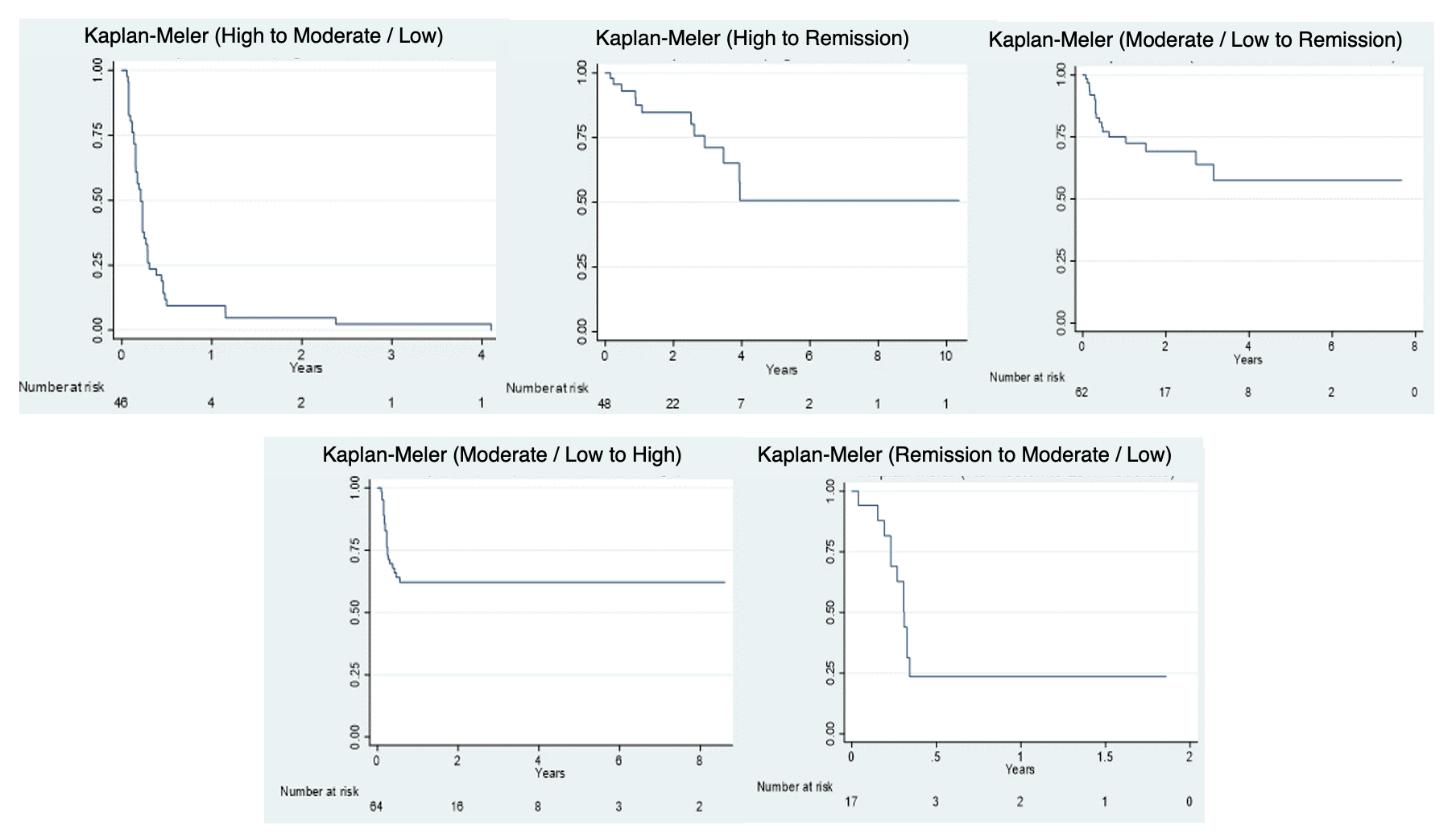

Supplement: Supplementary file 2 — Additional file 2. Survival analysis. [file 12913_2023_9595_MOESM2_ESM.docx]
