# Supplementary material for: Cost-utility analysis of biologic disease-modifying antirheumatic drugs (bDMARDs), targeted synthetic DMARDs (tsDMARDs) and biosimilar DMARDs (bsDMARDs) combined with methotrexate for Thai rheumatoid arthritis patients with high disease activity
Source: BMC Health Serv Res. 2023 May 31;23:561. doi: 10.1186/s12913-023-09595-1 (PMC10230705; doi:10.1186/s12913-023-09595-1)
Supplement: Supplementary file 3 — Additional file 3. Inclusion criteria based on the PICOS framework. [file 12913_2023_9595_MOESM3_ESM.docx]

## Additional file 3. Inclusion criteria based on PICOS framework

| **Populations** | Active RA patients who were diagnosed according to the 1958, 1987, or 2010 classification criteria and were MTX-inadequate responders |
| --- | --- |
| **Interventions** | - Originator bDMARDs and their biosimilars alone or in combination with MTX or any other csDMARDs - anti-TNF-alpha - anti-interleukin-6 (IL-6) - anti-CD20 - Originator tsDMARDs alone or in combination with MTX or any other csDMARDs - JAK inhibitor |
| **Comparators** | Any medications that could be linked within the network |
| **Outcomes** | Efficacy based on DAS28-ESR criteria at 6, 12 and 24 months   - The proportion of RA patients with high DA who achieved remission (H2R) based on DAS28-ESR < 2.6 - The proportion of RA patients with high DA who achieved low to moderate DA (H2M) based on DAS28-ESR > 2.6 to 5.1 - The proportion of RA patients with low to moderate DA who achieved remission (M2R) based on DAS28-ESR < 2.6   Safety - Number of patients who experienced serious infection |
| **Study design** | RCTs of at least 24 weeks’ duration (excluding post-hoc analyses of RCTs and extension trials with treatment switching trials prior to week 24) |
