# Supplementary material for: Cost-utility analysis of biologic disease-modifying antirheumatic drugs (bDMARDs), targeted synthetic DMARDs (tsDMARDs) and biosimilar DMARDs (bsDMARDs) combined with methotrexate for Thai rheumatoid arthritis patients with high disease activity
Source: BMC Health Serv Res. 2023 May 31;23:561. doi: 10.1186/s12913-023-09595-1 (PMC10230705; doi:10.1186/s12913-023-09595-1)
Supplement: Supplementary file 4 — Additional file 4. Main findings of the SR-NMA. [file 12913_2023_9595_MOESM4_ESM.docx]

## Additional file 4. Main findings of SR-NMA

### *Additional file* *4.1. Relative risk with 95%CI of the efficacy outcomes for each treatment option in combination with MTX, arranged by health states and outcome measuring time points.*

| **Treatment options** | **Efficacy** | | | | **Safety** | |
| --- | --- | --- | --- | --- | --- | --- |
|  | **High to Remission** | | **High to Moderate / Low** | | **Serious infection** | |
|  | **6 months** | **12 months** | **6 months** | **12 months** | **6 months** | **12 months** |
| MTX | **Reference** | | | | | |
| ETA 25 mg + MTX | **2.23**  (1.64, 3.04) | **1.93**  (1.14, 3.25) | **3.00**  (1.77, 5.07) | - | - | - |
| IFX 3 mg/kg + MTX | **1.95**  (1.11, 3.43) | **1.75**  (1.06, 2.88) | - | - | - | - |
| GOL 50 mg + MTX | **2.96**  (2.03, 4.32) | - | - | - | **1.1**  (0.26, 4.63) | - |
| TCZ 8 mg/kg + MTX | **3.06**  (2.27, 4.12) | **2.52**  (1.94, 3.28) | - | - | - | **1.52**  (0.52, 4.49) |
| RTX 1,000 mg + MTX | **1.58**  (1.04, 2.40) | **2.38**  (1.65, 3.45) | **2.66**  (1.09, 6.46) | **2.15**  (1.61, 2.86) | **0.9**  (0.28, 2.86) | **0.61**  (0.22, 1.68) |
| TOF 5 mg + MTX | **2.49**  (1.70, 3.65) | **0.86**  (0.56, 1.33) | **2.14**  (1.63, 2.81) | **1.41**  (1.01, 1.96) | - | - |
| BAR 4 mg + MTX | **2.71**  (2.13, 3.44) | **1.67**  (1.17, 2.37) | **1.86**  (1.39, 2.48) | **1.67**  (0.98, 2.85) | **1.08**  (0.56, 2.10) | **1.47**  (0.53, 4.08) |
| bsIFXr (CT-P13) 3 mg/kg + MTX | **2.62**  (1.25, 5.46) | - | - | - | - | - |
| bsIFXi (PF-06438179/GP1111) 3mg/kg + MTX | **2.25**  (1.17, 4.33) | - | - | - | - | - |
| bsADA (ABP 501) 40 mg + MTX | **2.22**  (1.58, 3.12) | - | - | - | - | - |
| bsRTX (CT-P10) 1000 mg + MTX | **1.22**  (0.60, 2.51) | **1.71**  (1.02, 2.88) | - | **2.33**  (1.55, 3.49) | - | **0.4**  (0.05, 3.06) |

Note: “-” indicates no data

MTX: Methotrexate, bs: biosimilar, ETA: Etanercept (Enbrel^®^), IFX: Infliximab (Remicade^®^), GOL: Golimumab (Simponi^®^), TCZ: Tocilizumab (Actemra^®^), RTX: Rituximab (Mabthera^®^), TOF: Tofacitinib (Xeljanz^®^), BAR: Baricitinib (Olumiant^®^), bsIFXr: Biosimilar infliximab (Remsima^®^), bsIFXi: Biosimilar infliximab (Ixifi^®^), bsADA: Biosimilar adalimumab (Amgevita^®^), bsRTX: Biosimilar rituximab (Truxima^®^)

### *Additional file 4.2. Relative risk with 95%CI of assumed efficacy employed in Markov model*

| **Treatment options** | **High to Remission** | | | **Moderate / Low to Remission** | | | **High to Moderate / Low** | | | **Safety** | |
| --- | --- | --- | --- | --- | --- | --- | --- | --- | --- | --- | --- |
|  |  |  |  |  |  |  |  |  |  | **Serious infection** | |
|  | **6 months** | **12 months** | **24 months** | **6 months** | **12 months** | **24 months** | **6 months** | **12 months** | **24 months** | **6 months** | **12 months** |
| **MTX** | **Reference** | | | | | | | | | | |
| **ETA 25 mg + MTX** | **2.23**  (1.64, 3.04) | **1.93**  (1.14, 3.25) | **1.93**  (1.14, 3.25) | **2.23**  (1.64, 3.04) | **1.93**  (1.14, 3.25) | **1.93**  (1.14, 3.25) | **3.00**  (1.77, 5.07) | - | - | - | - |
| **IFX 3 mg/kg + MTX** | **1.95**  (1.11, 3.43) | **1.75**  (1.06, 2.88) | **1.75**  (1.06, 2.88) | **1.95**  (1.11, 3.43) | **1.75**  (1.06, 2.88) | **1.75**  (1.06, 2.88) | - | - | - | - | - |
| **GOL 50 mg + MTX** | **2.96**  (2.03, 4.32) | **2.96**  (2.03, 4.32) | **2.96**  (2.03, 4.32) | **2.96**  (2.03, 4.32) | **2.96**  (2.03, 4.32) | **2.96**  (2.03, 4.32) | - | - | - | **1.1**  (0.26, 4.63) | - |
| **TCZ 8 mg/kg + MTX** | **3.06**  (2.27, 4.12) | **2.52**  (1.94, 3.28) | **2.52**  (1.94, 3.28) | **3.06**  (2.27, 4.12) | **2.52**  (1.94, 3.28) | **2.52**  (1.94, 3.28) | - | - | - | - | **1.52**  (0.52, 4.49) |
| **RTX 1,000 mg + MTX** | **1.58**  (1.04, 2.40) | **2.38**  (1.65, 3.45) | **2.38**  (1.65, 3.45) | **1.58**  (1.04, 2.40) | **2.38**  (1.65, 3.45) | **2.38**  (1.65, 3.45) | **2.66**  (1.09, 6.46) | **2.15**  (1.61, 2.86) | - | **0.9**  (0.28, 2.86) | **0.61**  (0.22, 1.68) |
| **TOF 5 mg + MTX** | **2.49**  (1.70, 3.65) | **0.86**  (0.56, 1.33) | **0.86**  (0.56, 1.33) | **2.49**  (1.70, 3.65) | **0.86**  (0.56, 1.33) | **0.86**  (0.56, 1.33) | **2.14**  (1.63, 2.81) | **1.41**  (1.01, 1.96) | - | - | - |
| **BAR 4 mg + MTX** | **2.71**  (2.13, 3.44) | **1.67**  (1.17, 2.37) | **1.67**  (1.17, 2.37) | **2.71**  (2.13, 3.44) | **1.67**  (1.17, 2.37) | **1.67**  (1.17, 2.37) | **1.86**  (1.39, 2.48) | **1.67**  (0.98, 2.85) | - | **1.08**  (0.56, 2.10) | **1.47**  (0.53, 4.08) |
| **bsIFXr (CT-P13) 3 mg/kg + MTX** | **2.62**  (1.25, 5.46) | **2.62**  (1.25, 5.46) | **2.62**  (1.25, 5.46) | **2.62**  (1.25, 5.46) | **2.62**  (1.25, 5.46) | **2.62**  (1.25, 5.46) | - | - | - | - | - |
| **bsIFXi (PF-06438179/GP1111) 3mg/kg + MTX** | **2.25**  (1.17, 4.33) | **2.25**  (1.17, 4.33) | **2.25**  (1.17, 4.33) | **2.25**  (1.17, 4.33) | **2.25**  (1.17, 4.33) | **2.25**  (1.17, 4.33) | - | - | - | - | - |
| **bsADA (ABP 501) 40 mg + MTX** | **2.22**  (1.58, 3.12) | **2.22**  (1.58, 3.12) | **2.22**  (1.58, 3.12) | **2.22**  (1.58, 3.12) | **2.22**  (1.58, 3.12) | **2.22**  (1.58, 3.12) | - | - | - | - | - |
| **bsRTX (CT-P10) 1000 mg + MTX** | **1.22**  (0.60, 2.51) | **1.71**  (1.02, 2.88) | **1.71**  (1.02, 2.88) | **1.22**  (0.60, 2.51) | **1.71**  (1.02, 2.88) | **1.71**  (1.02, 2.88) | - | **2.33**  (1.55, 3.49) | - | - | **0.4**  (0.05, 3.06) |

Note: Cells shaded yellow indicate an assumed efficacy, “-” indicates no data

MTX: Methotrexate, bs: biosimilar, ETA: Etanercept (Enbrel^®^), IFX: Infliximab (Remicade^®^), GOL: Golimumab (Simponi^®^), TCZ: Tocilizumab (Actemra^®^), RTX: Rituximab (Mabthera^®^), TOF: Tofacitinib (Xeljanz^®^), BAR: Baricitinib (Olumiant^®^), bsIFXr: Biosimilar infliximab (Remsima^®^), bsIFXi: Biosimilar infliximab (Ixifi^®^), bsADA: Biosimilar adalimumab (Amgevita^®^), bsRTX: Biosimilar rituximab (Truxima^®^)
